# Second primary malignancy in patients with esophageal adenocarcinoma and squamous cell carcinoma

**DOI:** 10.1097/MD.0000000000017083

**Published:** 2019-09-06

**Authors:** Guoqing Zhang, Bin Wu, Xiaofei Wang, Jindong Li

**Affiliations:** Department of thoracic surgery, First Affiliated Hospital of Zhengzhou University, Zhengzhou, Henan Province, China.

**Keywords:** competing risk, esophageal cancer, nomogram, second primary malignancy, SEER

## Abstract

There have been no studies on implementing effective screening models for esophageal adenocarcinoma and squamous cell carcinoma survivors. We used a proportional subdistribution hazards model to estimate second primary malignancy risks among patients with esophageal adenocarcinoma and squamous cell carcinoma. We validated models using a bootstrap cross-validation method and performed decision curve analysis to evaluate their clinical utility. Age group and SEER historic stage were significantly associated with second primary malignancy risk after diagnosis of esophageal adenocarcinoma and squamous cell carcinoma. Saving positive lymph nodes and distant metastasis were significant factors in the adenocarcinoma group, and marital status, tumor location, and chemotherapy were significant factors in the squamous cell carcinoma group. Calibration plots show good concordance between predicted and actual outcomes except in high-probability areas for the risk of a second primary malignancy in patients with esophageal squamous cell carcinoma. Discrimination performances of the Fine–Gray models were evaluated using c-indices, which were 0.691 and 0.662 for second primary malignancies in patients with esophageal adenocarcinoma and squamous cell carcinoma, respectively. Decision curve analysis yielded a range of threshold probabilities (0.020–0.177 and 0.021–0.133 for patients with esophageal adenocarcinoma and squamous cell carcinoma, respectively) at which the clinical net benefit of the risk model was larger than those of hypothetical all-screening and no-screening scenarios. Our nomograms enable selection of patient populations at high risk for a second primary malignancy and thus will facilitate the design of prevention trials for affected populations.

## Abstract 2

Supplemental Digital Content is available in the text

## 1 Introduction

Esophageal cancer, with an estimated 455,800 new cases and 400,200 deaths in 2012 worldwide,^[1]^ is a highly prevalent malignancy. From 2000 to 2014, the 5-year age-standardized net survival, from global surveillance data, was in the range 10% to 30%.^[2]^ Due to the poor prognosis for those with esophageal cancer, studies regarding the risk of developing a second primary malignancy (SPM) in these patients are relatively scarce. In recent years, the incidence of esophageal cancer has tended to decrease according to the Surveillance, Epidemiology and End Results (SEER) registry, and survival times have tended to increase for both early-stage and late-stage diseases.^[2,3]^ Combined with other facts such as the advancement of early detection techniques, surgical procedures, and perioperative adjuvant therapies, the number of long-term cancer survivors continues to increase, which has resulted in an increased incidence of SPMs in patients with esophageal cancer. Previous studies have shown that 4.0% to 37.4% of esophageal cancer survivors developed a SPM during their follow-up period,^[4–11]^ which will result in an even heavier burden related to the survival of patients with esophageal cancer.

Several previous studies have shown that screening models are important to efficiently detect primary esophageal cancer (PEC).^[12–14]^ To develop an effective screening nomogram for evaluating the probability of developing a SPM in patients with esophageal cancer, it is essential to identify associated risk factors. However, despite the increasing importance of SPMs, there currently are no consensus guidelines for survivors of esophageal cancer who are at a relatively high risk of a SPM. Although several studies have evaluated the prevalence and risk of SPMs, most of them have tended to discuss the standardized incidence ratios (SIRs) of SPMs or the risk factors associated with SPMs, rather than providing prediction models for survivors of PEC.^[7–9,15]^ Furthermore, few published studies have taken into account competing risks, leading to a substantial bias in risk estimation of SPMs.^[16]^

Adenocarcinoma (AC) and squamous cell carcinoma (SCC) are the 2 main types of esophageal cancer. In this study, we developed and validated prediction nomograms for SPM risk in patients with esophageal AC and SCC on the basis of the demographic, diagnostic, and treatment factors using data from the SEER database.

## 2 Materials and methods

### 2.1 Data sources and case selection

Data were obtained from the 9 newest population-based cancer registries (Atlanta (Metropolitan), Connecticut, Detroit (Metropolitan), Hawaii, Iowa, New Mexico, San Francisco-Oakland SMSA, Seattle (Puget Sound) and Utah) (1973–2014) for this large population-based study. The data collected included demographic information (age, sex, race, and marital status), diagnostic information (tumor location, detailed information from the 8th edition American Joint Committee on Cancer (AJCC) staging system, SEER historic stage, and grade), and treatment information (surgery, radiation, and chemotherapy).

This study was deemed exempt from review by the Zhengzhou University Institutional Review Boards.

### 2.2 Statistical analysis

Kaplan–Meier survival and Cox model analyses tend to overestimate risks and reduce predictions regarding survival.^[16–18]^ Therefore, we employed the Fine–Gray model to estimate the unbiased risks in the presence of competing risks, and we regarded a SPM and death prior to developing a SPM as 2 competing events in our Fine–Gray model analysis. The cumulative incidence function (CIF) was used to show the probability of each event and Gray test was used to estimate the differences in CIFs between the groups. We developed competing-risk nomograms using the Fine–Gray model to predict SPM risk in patients with esophageal AC and SCC. Furthermore, we validated the nomograms using a bootstrap cross-validation method (200 bootstrap resamples). The c-index measures the discrimination ability of the model. Calibration plots were used to assess the predicted probabilities from the model vs the actual probabilities. Decision curve analysis was used to estimate clinical usefulness and net benefits. All differences were considered statistically significant if the 2-sided *P* value was <.05.

Data were analyzed with STATA software V.12.0 and R version 3.4 (http://www.r-project.org/).

## 3 Results

**Figure 1:**
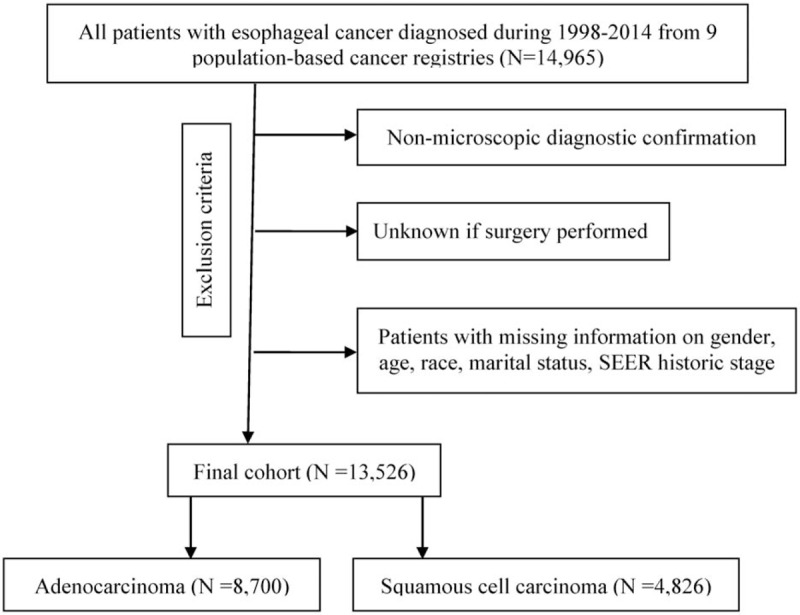
The subject selection algorithm. SEER: Surveillance, Epidemiology and End Results database.

We extracted the data of 13,526 patients who were diagnosed with esophageal AC (8,700 patients) or SCC (4,826 patients) from 1998 to 2014 with strict inclusion criteria (Fig. 1). There were 817 patients diagnosed with a SPM during the follow-up period: 480 patients in the AC group and 337 patients in the SCC group. Baseline characteristics of the cohort are summarized in supplementary Table 1. The maximum follow-up was 203 months in both the esophageal AC and SCC groups, and the median follow-up times were 11 and 8 months from the diagnosis of PEC in the AC and SCC groups, respectively.

Results for the Fine–Gray model are listed in Table 1, which shows different risk factors between the AC and SCC groups. Our results indicated that the age group and SEER historic stage were significantly associated with SPM risk in patients with esophageal AC and esophageal SCC. Saving positive lymph nodes and distant metastasis were significant risk factors in the AC group, and marital status, tumor location, and chemotherapy were significant factors in the SCC group. For example, from the model for the AC group, we can conclude the following:

1. Older patients (55–79 years) had substantially increased risks of SPMs, with a subdistribution hazard ratio (sHR) > 1.64 compared to that of patients 0 to 54 years old.
2. Compared to patients with negative lymph nodes, patients with 3 to 6 positive or unknown lymph nodes had significantly decreased risks of SPMs (sHRs = 0.49, *P* = .022 and sHRs = 0.69, *P* = .045, respectively).

**Table 1:**
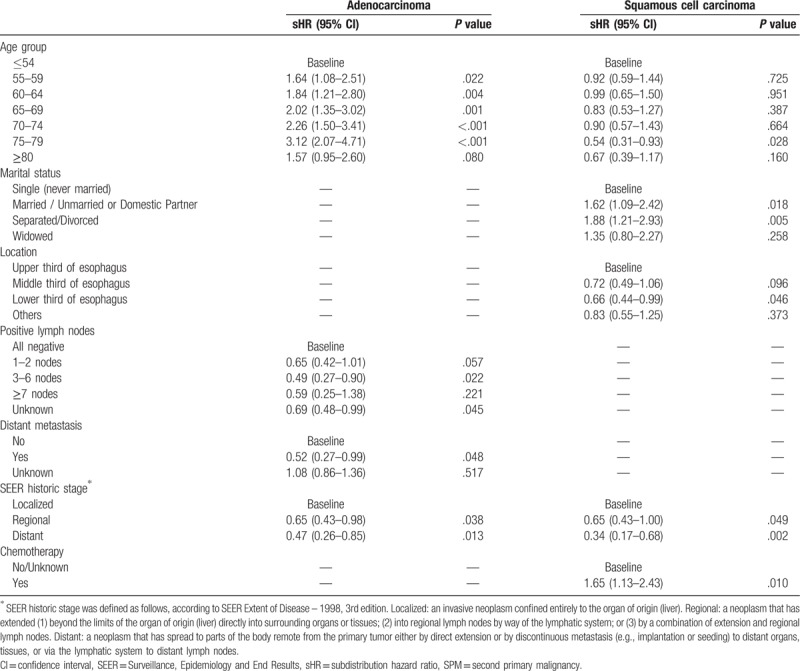
Association of the selected factors with SPM in the final prediction model for SPM risk in patients with adenocarcinoma and squamous cell carcinoma.

Patients with distant metastasis had significantly reduced risks compared to the risks of those without metastasis, with a sHR < 0.52. Patients with a regional or distant tumor had significantly reduced risks compared to the risks of those with a localized tumor, with a sHR < 0.65.

**Figure 2:**
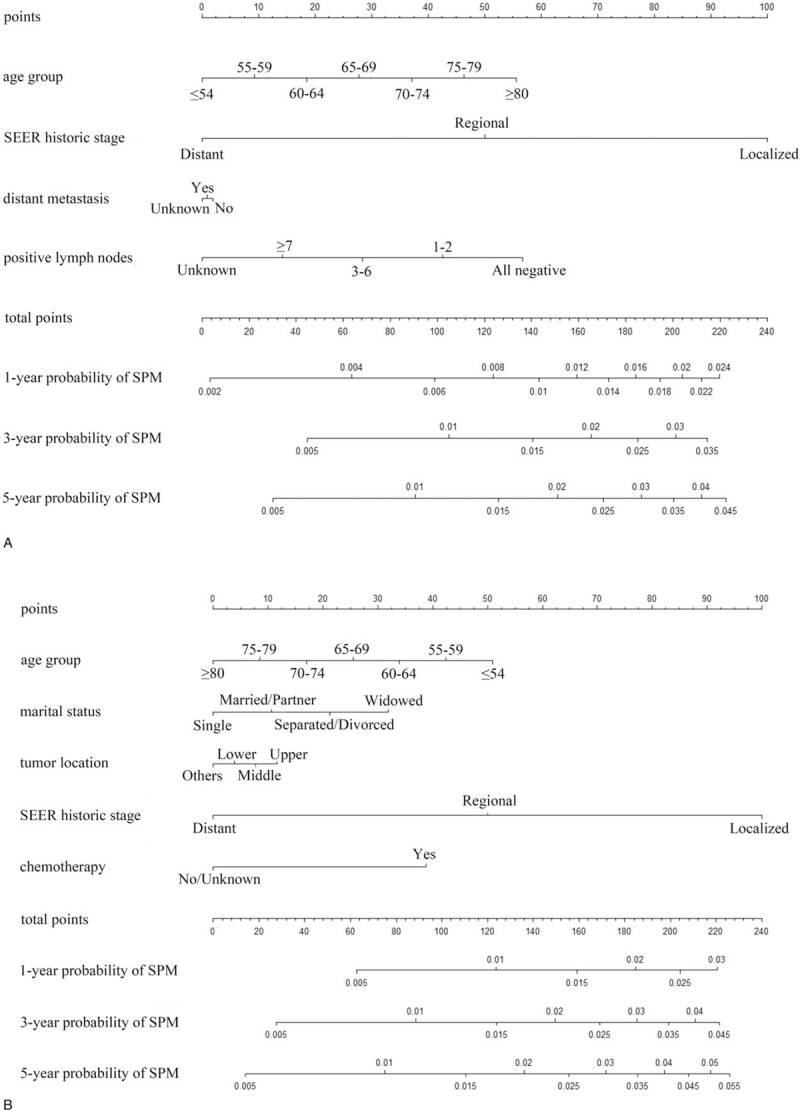
The nomograms for predicting SPM risks in patients with esophageal AC (A) and SCC (B). AC, adenocarcinoma; SCC, squamous cell carcinoma; SPM, second primary malignancy.

**Figure 3:**
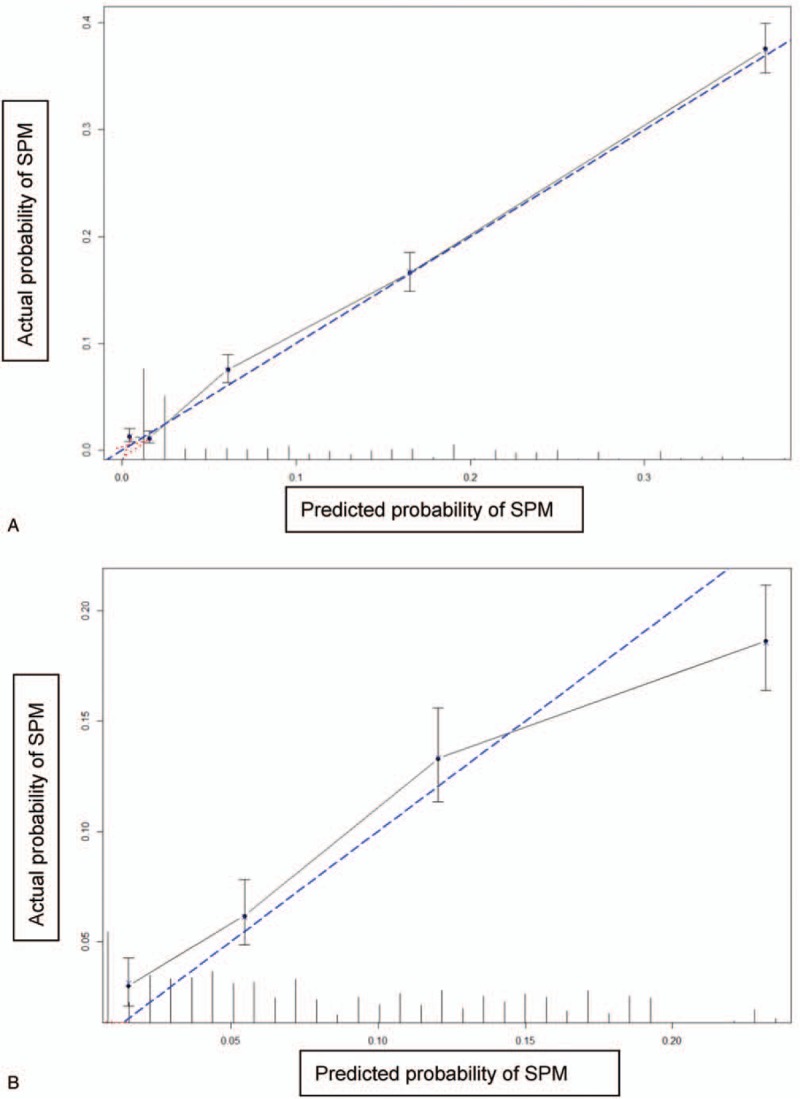
Calibration plots of the nomograms for predicting SPM in patients with esophageal AC (A) and SCC (B). AC, adenocarcinoma; SCC, squamous cell carcinoma; SPM, second primary malignancy.

**Figure 4:**
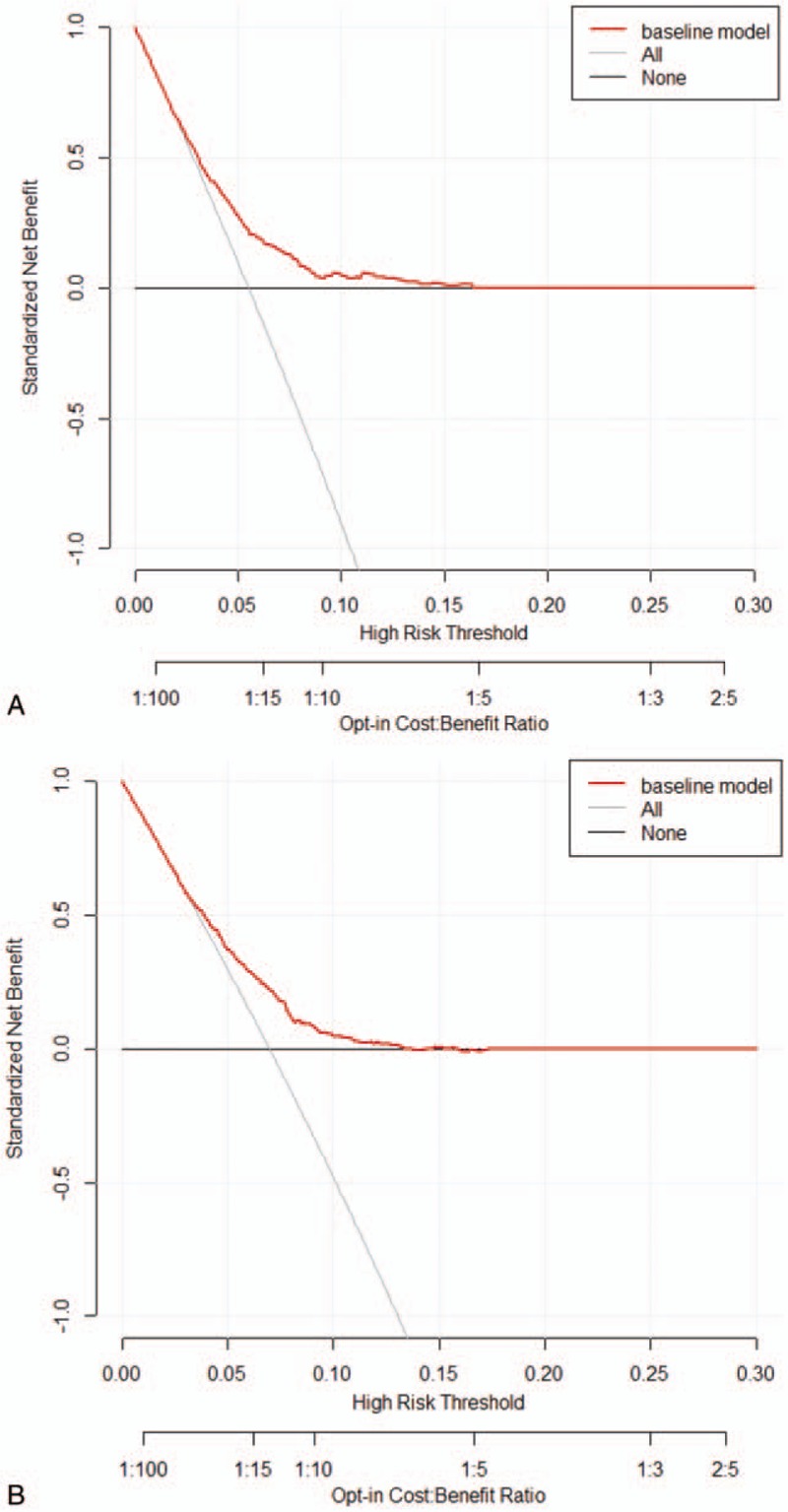
Decision curve analysis for the risk models for SPM in patients with esophageal AC (A) and SCC (B). AC, adenocarcinoma; SCC, squamous cell carcinoma; SPM, second primary malignancy.

Figure 2 shows the nomograms for predicting SPM risks in patients with esophageal AC and SCC. The calibration plots demonstrated good concordance between the predicted and actual outcomes (Fig. 3) except in high-probability areas for the risk of a SPM in patients with esophageal SCC (Fig. 3B). Furthermore, the discrimination performance of the Fine–Gray models were evaluated using the c-indices, which had values of 0.691 and 0.662 for SPMs in patients with esophageal AC and SCC, respectively. Finally, we compared the net benefits of the Fine–Gray models to those for 2 alternative scenarios: screening all individuals and screening no one. The results shown in Figure 4 demonstrate that the net benefits obtained from the application of the Fine–Gray models were higher than those in hypothetical all-screening or no-screening scenarios, as threshold probabilities were 0.020 to 0.177 and 0.021 to 0.133 in patients with esophageal AC and SCC, respectively. This implies that if we use a risk threshold from the above given intervals, such that screening is recommended if an individual's risk is above the given threshold, then the calculated net benefit is larger for the prediction model than it is for the strategies that do not use the model.

## 4 Discussion

As far as we know, this is the first study to develop nomograms for predicting the probability of developing a SPM in patients with esophageal AC and SCC using data from a large population-based cohort. As mentioned previously, we applied competing risk models to obtain unbiased estimates of the risk of SPMs. Our findings show that age group and SEER historic stage are significantly associated with SPM risk in patients with esophageal AC and SCC. Saving positive lymph nodes and distant metastasis were significant factors in the AC group, and marital status, tumor location, and chemotherapy were significant factors in the SCC group. Furthermore, the nomograms were well fitted to the ideal line of the calibration plot (Fig. 3) except in high-probability areas for the risk of a SPM in patients with esophageal SCC (Fig. 3B). The discrimination of our nomograms showed 69.1% and 66.2% accuracy for developing a SPM in patients with esophageal AC and SCC, respectively.

Previous studies have examined the relationship of age with the relative risk of SPMs, but the conclusions were not definitive. In general, it is known that the risk of SPMs tends to be higher for cancer patients diagnosed at a younger age^[19–21]^ because younger survivors have more time to develop a SPM. However, studies have reported an association between a younger age and lower risks of SPMs.^[22,23]^ Our study found different conclusions between patients with esophageal AC and those with esophageal SCC. Older patients (55–79 years) had a significantly higher risk of a SPM for those with patients with esophageal AC. However, the risk of a SPM tended to decline with advancing age (75–79 years) in patients with esophageal SCC. As reported previously, gastroesophageal acid reflux is an important risk factor for developing AC, while smoking is a strong risk factor for developing SCC.^[24]^ It is possible that our conclusions exist partly because patients with esophageal cancer have different underlying diseases or smoking habits. Our results suggest that physicians should monitor a range of data about environmental and lifestyle factors, as well as other potentially important information.

The emergence of SPMs in patients with esophageal cancer was once thought to be a risk factor of a poor prognosis. However, studies have shown that the outcomes of patients with SPMs are not necessarily poor.^[25]^ In our study, we found better survival in patients with SPMs (Supplementary Fig. 1). The reason for this may be that patients with a better prognosis suffered a higher incidence of SPMs due to their longer follow-up durations, possibly resulting from radical treatment. To summarize the information in Table 1, factors associated with better prognosis include the absence of lymph node metastasis or distant metastasis in patients with esophageal AC, localized tumors in patients with esophageal AC and SCC, and chemotherapy in patients with esophageal SCC. Interestingly, we found that single patients tend to have lower risks of developing SPMs (Table 1), which is likely due to the fact that single patients had worse survival outcomes than married patients.^[26]^ For tumor location, the different risks were likely related to different surgical difficulties; for example, upper thoracic esophageal cancer is more difficult to resect than lower esophageal cancer. Currently, there is much controversy related to procedural complexity.^[25,27–30]^ Several studies have reported that middle third esophageal cancer has a statistically worse prognosis than upper third esophageal cancer,^[27,28,30]^ which may result in a lower risk of SPMs in patients with middle third esophageal cancer, which is consistent with the results of our study.

There are currently no optimal treatment algorithms for patients with esophageal cancer who develop SPMs. As reported, chemoradiation therapy is an important treatment option for these patients. However, there is evidence that the risk of developing SPMs also increases after chemoradiation therapy,^[31]^ which is partly consistent with the results of our study. As shown in Table 1, patients with esophageal SCC had a significantly increased risk (sHR = 1.65, *P* = .010), compared to that of patients with no/unknown chemotherapy treatment. Chemoradiation therapy increases the risk of SPMs in patients with esophageal SCC because many agents used in chemotherapy, as well as ionizing radiation, are known carcinogens, which induce genetic mutations and immune system alterations.^[32]^ From the present point of view, all cancers should be treated with a curative intent, and SPMs often require extremely complex and more invasive surgical procedures to resect all affected regions curatively. Otowa et al^[33]^ recommended that surgery should be selected as a first-line treatment in patients with SPMs after diagnosis of esophageal SCC. Furthermore, as our research indicates, surgery did not increase the incidence of SPMs in patients with esophageal cancer (Table 1). Therefore, intense screening to detect curable SPMs and surgery to treat curable SPMs should be justified.

Several limitations of our study exist, some of which have been discussed in our previous study.^[34]^ Because of these limitations, it is possible that the lack of sufficient data led to the moderate c-indices observed from our models. In addition, and most importantly, independent external validation to confirm efficacy and identify possible additional indices that might strengthen the mathematical basis of these predictions is needed.

In conclusion, we developed and validated predictive nomograms for SPM risk in patients with esophageal AC and SCC on the basis of clinical and demographic risk factors using data from a large population-based cohort. Our nomograms allow selection of a patient population at high risk for SPM and thus will facilitate the design of prevention trials for the affected population. However, further studies that include external validation and the extension of the proposed nomograms using various possible parameters as risk predictors are needed to provide enough evidence to justify our conclusions.

## Author contributions

**Conceptualization:** Guoqing Zhang, Bin Wu, Xiaofei Wang, Jindong Li.

**Data curation:** Guoqing Zhang, Bin Wu, Xiaofei Wang, Jindong Li.

**Formal analysis:** Guoqing Zhang, Bin Wu, Xiaofei Wang, Jindong Li.

**Funding acquisition:** Bin Wu, Xiaofei Wang, Jindong Li.

**Investigation:** Guoqing Zhang, Bin Wu, Xiaofei Wang, Jindong Li.

**Methodology:** Guoqing Zhang, Bin Wu, Xiaofei Wang, Jindong Li.

**Project administration:** Guoqing Zhang, Bin Wu, Xiaofei Wang, Jindong Li.

**Resources:** Guoqing Zhang, Bin Wu, Jindong Li.

**Software:** Guoqing Zhang, Bin Wu, Jindong Li.

**Supervision:** Jindong Li.

**Validation:** Guoqing Zhang, Jindong Li.

**Visualization:** Guoqing Zhang, Jindong Li.

**Writing – original draft:** Guoqing Zhang, Bin Wu.

## Supplementary Material

Supplemental Digital Content
