# Supplementary figures and images for: Second primary malignancy in patients with esophageal adenocarcinoma and squamous cell carcinoma
Source: Medicine (Baltimore). 2019 Sep 6;98(36):e17083. doi: 10.1097/MD.0000000000017083 (PMC6738979; doi:10.1097/MD.0000000000017083)

**Supplementary figure 1**

**
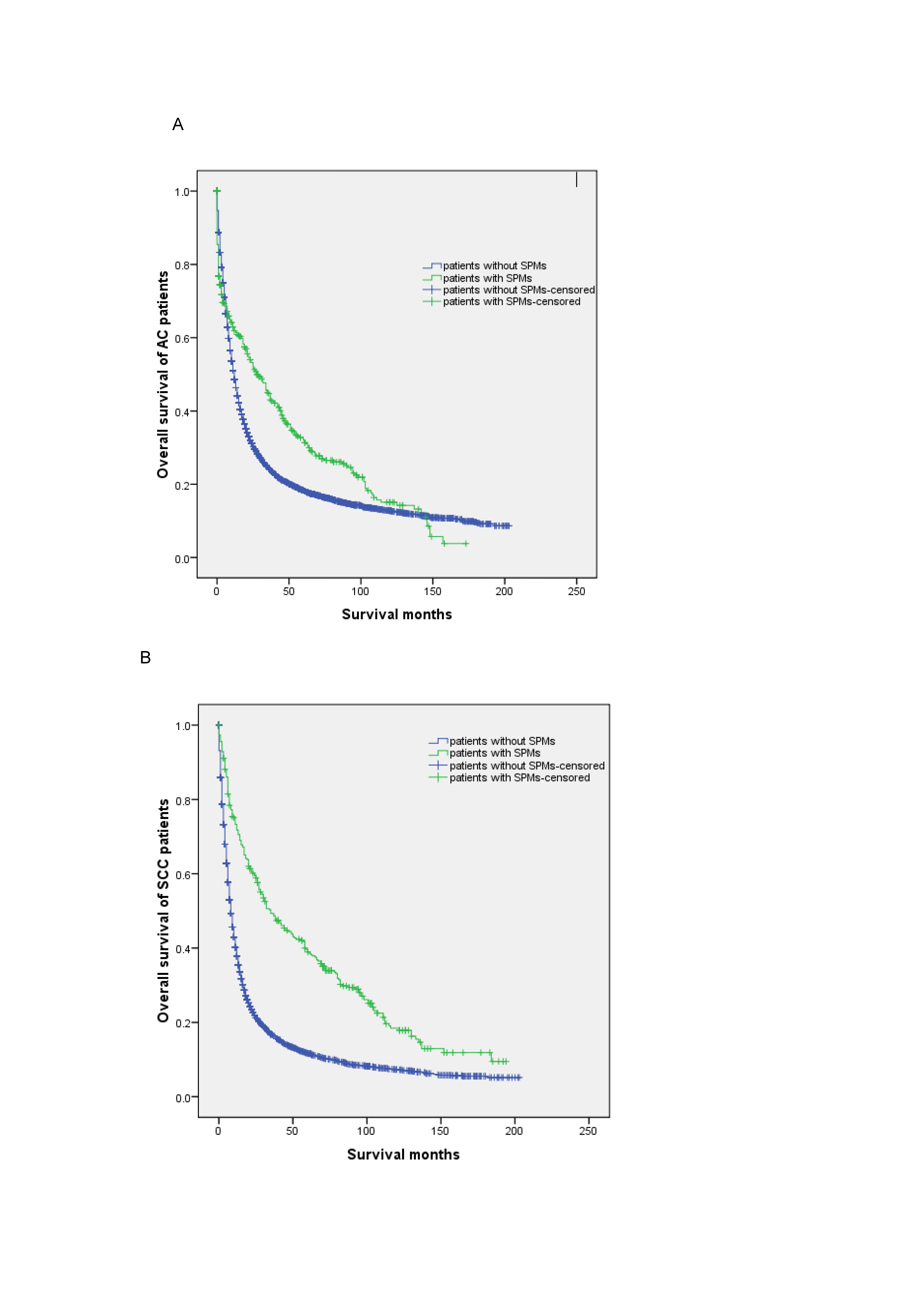
**

Supplement: Supplemental Digital Content [file medi-98-e17083-s001.docx]
